# Collagen Structure of Tendon Relates to Function

**DOI:** 10.1100/tsw.2007.92

**Published:** 2007-03-30

**Authors:** Marco Franchi, Alessandra Trirè, Marilisa Quaranta, Ester Orsini, Victoria Ottani

**Affiliations:** Department of Human Anatomical Sciences and Physiopathology of Locomotor Apparatus, University of Bologna, 48 via Irnerio, Bologna, IT 40126, Italy

**Keywords:** collagen, crimps, electron microscopy, fibrils, tendon, ultrastructure

## Abstract

A tendon is a tough band of fibrous connective tissue that connects muscle to bone, designed to transmit forces and withstand tension during muscle contraction. Tendon may be surrounded by different structures: 1) fibrous sheaths or retinaculae; 2) reflection pulleys; 3) synovial sheaths; 4) peritendon sheaths; 5) tendon bursae. Tendons contain a) few cells, mostly represented by tenoblasts along with endothelial cells and some chondrocytes; b) proteoglycans (PGs), mainly decorin and hyaluronan, and c) collagen, mostly type I. Tendon is a good example of a high ordered extracellular matrix in which collagen molecules assemble into filamentous collagen fibrils (formed by microfibrils) which aggregate to form collagen fibers, the main structural components. It represents a multihierarchical structure as it contains collagen molecules arranged in fibrils then grouped in fibril bundles, fascicles and fiber bundles that are almost parallel to the long axis of the tendon, named as primary, secondary and tertiary bundles. Collagen fibrils in tendons show prevalently large diameter, a D-period of about 67 nm and appear built of collagen molecules lying at a slight angle (< 5). Under polarized light microscopy the collagen fiber bundles appear crimped with alternative dark and light transverse bands. In recent studies tendon crimps observed via SEM and TEM show that the single collagen fibrils suddenly changing their direction contain knots. These knots of collagen fibrils inside each tendon crimp have been termed “fibrillar crimps”, and even if they show different aspects they all may fulfil the same functional role. As integral component of musculoskeletal system, the tendon acts to transmit muscle forces to the skeletal system. There is no complete understanding of the mechanisms in transmitting/absorbing tensional forces within the tendon; however it seems likely that a flattening of tendon crimps may occur at a first stage of tendon stretching. Increasing stretching, other transmission mechanisms such as an interfibrillar coupling via PGs linkages and a molecular gliding within the fibrils structure may be involved.

## INTRODUCTION

Tendons are white-brilliant anatomical structures interposed between muscles and bone. Showing a great resistance to mechanical loads[1] they make joint movement possible as they act as almost inextensible fibrous-elastic structures transmitting the forces generated by muscle contraction directly to the bone with a minimal dispersion of energy[2,3]. The greater the force generated by muscle is, the greater the stress transmitted through the tendon[4]. Tendons are very resistant and their potential loading is consistent: Carlstedt and Nordin[5] reported that during normal activity, a tendon *in vivo* is subjected to less than 25% of its tensile strength.

Tendons vary considerably in shape and size, ranging from flat to cylindrical, fan shaped and ribbon shaped tendons. The shape and properties of tendons are significantly related to the behaviour of the entire muscle-tendon complex[6]. Muscles creating powerful or resistive forces, like the quadriceps muscle, have short thick tendons, while those that have to perform soft or delicate movements, like the finger flexors, have long thin tendons[1].

Mammalian tendons are composed of cells and almost exclusively of extracellular collagen fibrils embedded in a proteoglycan/water extracellular matrix or ground substance[1,6,7,8,9]. Tendon is a good example of a highly ordered extracellular matrix in which collagen molecules assemble into filamentous collagen fibrils, formed by microfibrils[10,11,12,1314] which aggregate to form collagen fibers, the main structural components[8,15,16,17]. Tendon is also a good example of multihierarchical structure as it contains collagen molecules arranged in fibrils then grouped in fibril bundles, fascicles and fiber bundles that are almost parallel to the long axis of the tendon, named as primary, secondary and tertiary bundles (Fig. 1)[7]. Different terms were subsequently proposed for the collagen fiber bundles, because of their wide variability in different tendons[1].

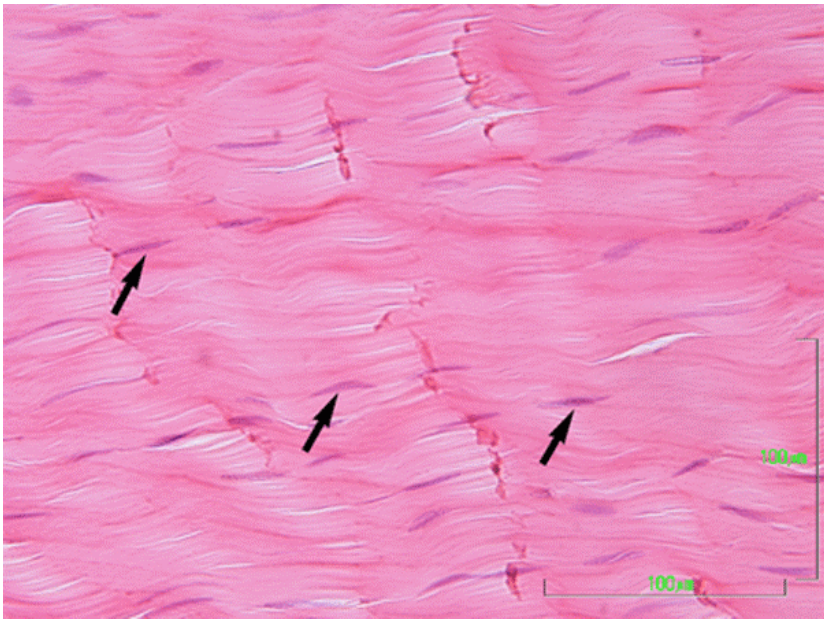

**FIGURE 1.** Light microscope image of longitudinally sectioned human Achilles tendon. Collagen fiber bundles appear parallel, wavyform with interposed flattened parallel fibroblasts (arrows).
Bar = 100 µm

## TENDON SHEATHS

Tendon may be surrounded by different structures: 1) fibrous sheaths or retinaculae; 2) reflection pulleys; 3) synovial sheaths; 4) peritendon sheaths (paratenon), in tendon with no synovial sheath; 5) tendon bursae[1].

When appreciable movement of tendon may cause friction against the surrounding tissues, tendon is often surrounded by a connective tissue that is called paratenon (Figs. 2-4). However variable tendon sheaths have been described in different tendons. Cohen and Kaplan[18] observed a parietal synovium and a second visceral synovium in human flexor tendons. Leonardi et al.[19] described three different sheaths in rat tail tendon: 1) paratenon, 2) peritenoneum and 3) endotenoneum. Strocchi et al.[20] found four sheaths in rat tail tendon: 1) the external paratendineum, a thick fibrous sheath covering the four groups of tendons around the vertebrae of the tail; 2) the epitendineum, a fibrous sheath surrounding each tendon group; 3) the peritendineum, enveloping each tendon; 4) the endotendineum, adhering to the fibers of the tendon. These authors distinguished thick straight bundles of collagen fibrils with straight microfibrillar arrangement and variable diameter (35-220 nm) in paratendineum and epitendineum, and thin bundles of small collagen fibrils with helical microfibrillar arrangement and uniform diameter (50 nm) in peritendineum and endotendineum. Gotoh et al.[21] divided the rat tail tendon sheath into three layers: 1) visceral, 2) parietal and 3) fibrous. Birk[22] found that when tendon architecture is immature types I and III small collagen fibrils are codistributed through the fascicles and their investments, while immature, tendon fascicles type III collagen is restricted to the outer sheaths. Kvist[23] found that paratenon is composed of type I and type III collagen fibrils and minor elastic fibrils in humans. This peritendinous tissue is lined on its inner surface by synovial cells[24,25] and acts as an elastic sleeve permitting the movement of tendon against the surrounding tissues[1,26]. Kannus[1] and Magnusson et al.[6] reported that under paratenon the entire tendon is surrounded by epitenon, the fine connective true sheath that immediately envelops the tendon. Lining cells observed in synovial tendon sheath and bursae of horse appear to be fibroblasts, some of them capable of phagocytosis [27].

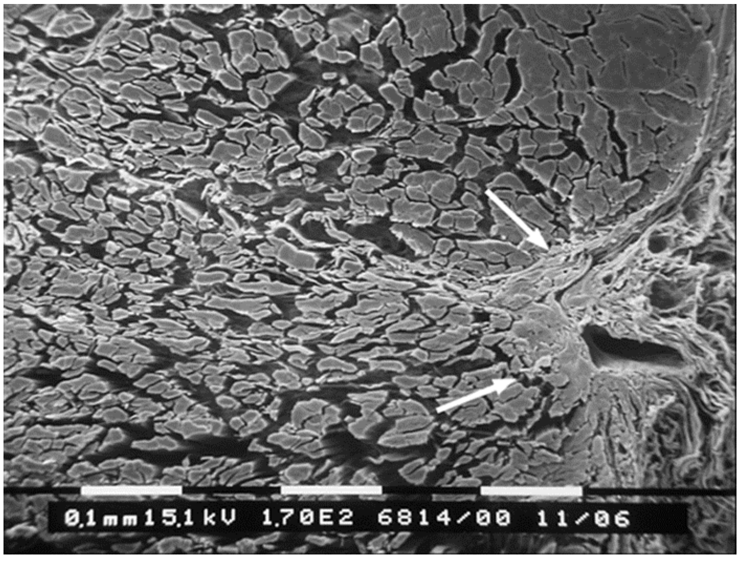

**FIGURE 2.** SEM image of transversally cut rat Achilles tendon. The polygonal collagen fascicles are clearly distinguishable. On the right paratenon is covering the surface of tendon and envelopes some blood vessels (arrows). From paratenon develops epitenon that runs deeply into the tendon. Bar = 100 µm

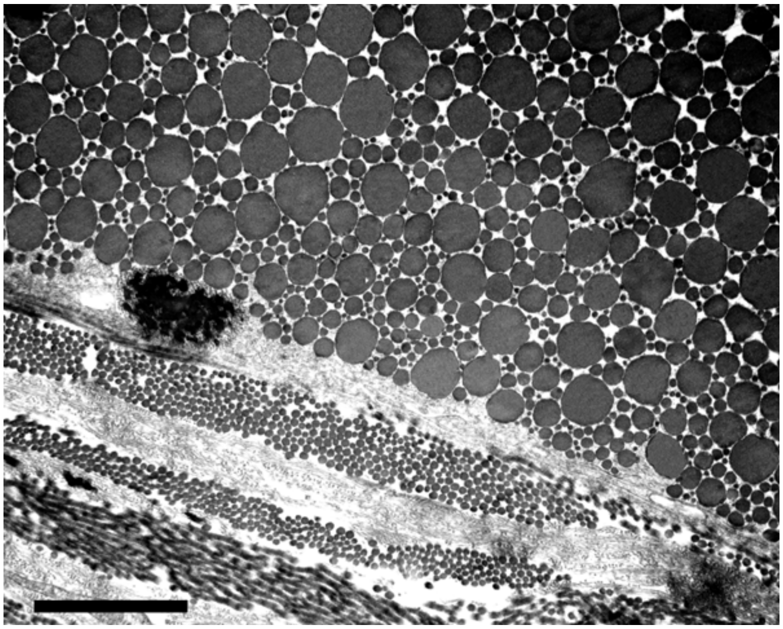

**FIGURE 3.** TEM section of rat Achilles tendon. Transversally sectioned tendon including plurimodal collagen fibrils (top) and unimodal fibrils of paratenon (bottom). Between tendon collagen fibers and paratenon elastic fibers are visible (dark stained).
Bar = 1 μm

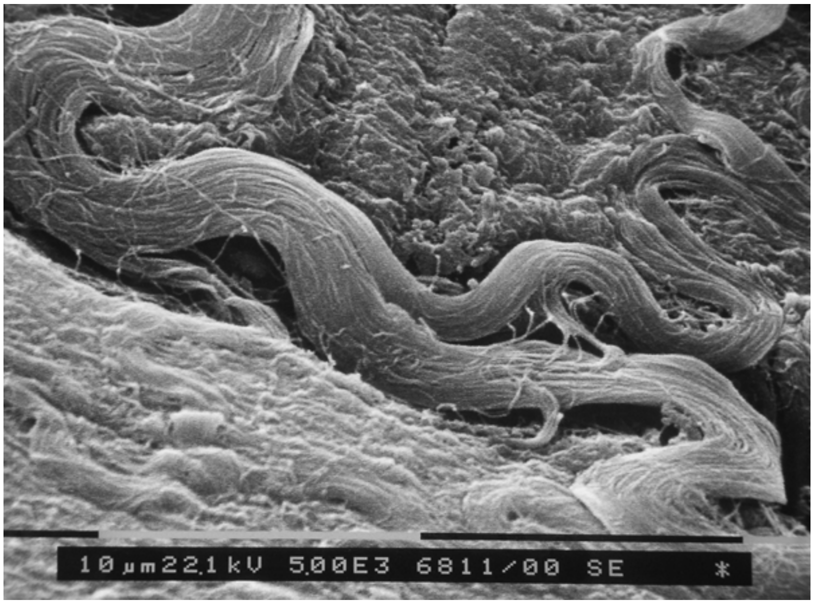

**FIGURE 4.** SEM image of rat Achilles tendon sheat. No fibrillar crimps are detectable when fibrils change their direction.
Bar= 10 μm

Schatzker and Branemark[28] observed that inside the tendon, collagen fibers and fiber bundles are enclosed in the endotenon, which serves to carry blood vessels, lymphatics and nerves. A further role of endotenon is correlated to the high degree of hydratation of proteoglycan components between the endotenon and the tendon fascicles[29,30]. These structures could allow the collagen fiber groups to glide on each other[1].

## CELLS

It is reasonable to think that the mechanical properties of tendon are dynamically dependent on the properties both of the cross-linked collagenous network and cell-extracellular matrix interactions [8].

Tendon cells are mostly (90-95%) represented by tenoblasts (fibroblasts) along with endothelial cells and some chondrocytes located in the areas of compression[1,6]. Tenoblasts have different morphology, some of them being elongated and others rounded[1]. The length of these cells varies from 20 µm to 70 µm and the width from 8µm to 20 µm[31]. Numerous long slender cytoplasmatic processes extend into the matrix[1] (Fig. 5). The number of tenoblasts decreases with aging and they turn into tenocytes of 80-300 µm in diameter with thin longer cellular processes[1].

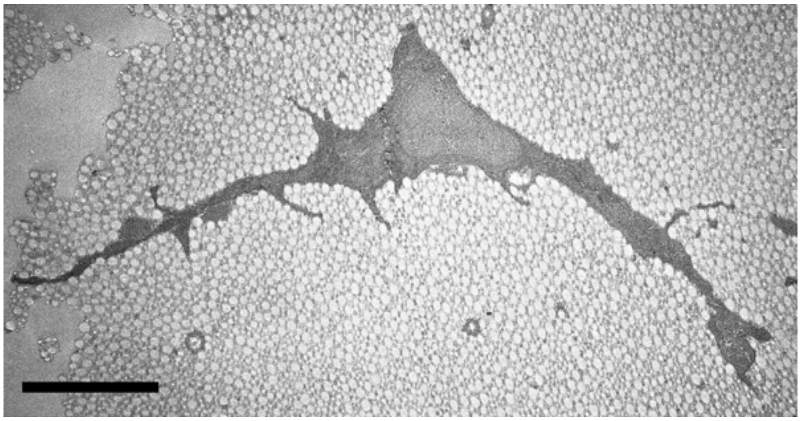

**FIGURE 5.** TEM image of cross-sectioned collagen fibrils and a tenocyte showing long and thin cytoplasmatic processes.
Bar = 2.5 µm

Fibroblasts synthesize molecules of the extracellular matrix such as collagen, proteoglycans and other proteins[9,32]. Collagen fibrils are produced in cellular recesses, then brought together to form collagen fibril bundles and the bundles are assembled into higher-order structures such as tendon fascicles[9]. Fibroblasts seem to influence collagen fibril orientation: collagen fibrils can be traced from locations deep within the cell, where they may coexist with numerous shorter fibrils, through a distinctive fibripositor (fibril-depositor) structure. This is located at the side of the cell, aligns along the long axis of the tendon and protrudes into the spaces between cells to extracellular collagen fibril bundles[9]. Fibripositors are absent at postnatal stages when collagen fibrils increase in diameter by accretion of extracellular collagen, but thereby maintaining parallelism of the tendon[33]. Collagen fibril proliferation seems to be influenced by mechanical strain in tendon[3,34]. Ingber[35] suggested that forces may be transmitted to and from cells through the extracellular matrix with changes in mechanical forces and cell shape acting as a biological regulator. It is well known that connective tissue cells adapt their extracellular matrix to changes in mechanical load and cells adhere to extracellular matrix via specialized cell surface receptors, the most important of them being integrins[36]. Integrin adhesion receptors have been implicated in the mechanism of signal transduction through the cell membrane in both directions[37,38]. Interactions between cells and extracellular matrix and exchanges between extracellular matrix and cells may be in a dynamic equilibrium[39]: *in vitro* studies demonstrated that internal (cytoskeletal) and external (elastic) forces are related via integrins if the substrate of fibroblast cultures is stretched or compressed[36].

## PROTEOGLYCANS

Tendon contains proteoglycans (PGs) like decorin and hyaluronan, but also biglycan, fibromodulin, lumican, epiphycan and keratocan[40,41]. PGs have been described as filaments regularly and orthogonally attached to the tendon collagen fibrils[42,43] (Fig. 6). PGs make up less than 1% of the dry weight of most tensile tendons[44]. The amount of PGs linked to the collagen fibrils in tendon decreases with increasing fibril diameter and age[45]. Decorin, biglycan, fibromodulin and lumican, all members of the small leucine-rich PG family, bind to collagen fibrils and are active participants in fibrillogenesis. Aggrecan and versican, two members of large modular PGs or lecticans, and their partner hyaluronan are

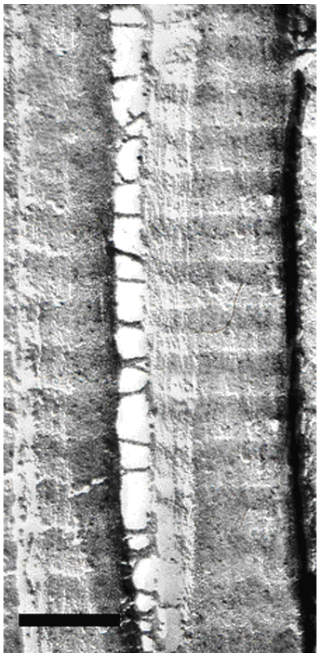

**FIGURE 6.** Orthogonally-oriented PGs interconnecting two collagen fibrils are clearly revealed by the freeze-etching process.
Bar = 0.2 μm likely to provide tendon tissues with a high capacity to resist high compressive and tensile forces associated with loading and mobilization[46]. PGs like decorin or other glycoproteins in extracellular matrix of tendon are related to collagen fibrillogenesis[8]. Decorin is a small interstitial dermatan sulphate PG and is specifically associated with the surface of fibrillar type I collagen in extracellular matrices[47,48]. Decorin seems to be related to the alignment, orienting and ordering of the collagen fibrils and facilitates the sliding of collagen fibrils during mechanical deformation[49,50]. During collagen fibrillogenesis, proteoglycans play an important role in guiding and stabilizing collagen fibril formation and maturation, preventing the aberrant lateral fusion of collagen fibrils[17,49,51,52,53,54,55]. In fact, in tendon fibromodulin may be required early in collagen fibrillogenesis to stabilize small-diameter fibril-intermediates and lumican may be needed at a later stage, primarily to limit lateral growth of fibrils[56]. Moreover, PGs extending from one collagen fibril to another at intervals of ~60 nm may play a role in transmitting and resisting tensile stresses in tendon during stretching[43]. Large PGs, such as aggrecan, are located in compressed regions of tendons; in fact aggrecan is the predominant PG in the extracellular matrix of tendon fibrocartilage where it provides a viscous environment that allows the collagen fibers to stretch and dissipate the force of sudden loads[48].

## COLLAGEN FIBERS

Collagen is the most abundant protein in mammalians and is composed of fibrils resulting from the aggregation of a triple helix of three polypeptide chains, 300 nm in length and 1.5 nm in diameter[57,58]. Several types of collagen have been described, but tendons are mainly composed of type I collagen, even if type II, III, V, XI have also been described[15,59,60]. Type I collagen, a co-polymer of two or more fibril-forming collagens, is synthetized as soluble procollagen chains within intracellular vesicles[60,61] and released from cell membrane invaginations into the extracellular matrix[8,9]. Then the chains undergo the removal of the globular C- and N- terminal propeptides by matrix metalloproteinases C-proteinase and N-proteinase, respectively, according to a key process that results in spontaneous collagen fibril formation[9,62]. Lateral fusion of fibril intermediates results in both linear and lateral growth of fibrils[8]. In tendons a bunch of parallel collagen fibrils forms a collagen fiber, aligned from end to end in a tendon[63], which is the smallest unit that can be tested mechanically[64]. There is no standard nomenclature for aggregations of collagen fibrils within tendon, perhaps because of their great variability[1]. However Kannus[1] suggested a nomenclature for aggregations of collagen fibrils: a bunch of collagen fibers forms a primary bundle or subfascicle (15-400 µm in diameter), and a group of primary bundles forms secondary fiber bundles or fascicles whose diameter range from 150 to 1000 µm in human tendons (Fig. 7). Silver et al.[59] reported that collagen fibril bundles in mature tendon have diameters ranging from 1 to 300 µm. A group of secondary fiber bundles forms a tertiary bundle (diameter from 1000 to 3000 µm in human tendons), which makes up the tendon surrounded by epitenon. Kannus[1] showed that three or four subfascicles form one fascicle, while Kastelic et al.[65] reported that tendon fascicles may have up to 10-12 fascicles. It seems that the number and the diameters of the subfascicles and fascicles vary from tendon to tendon and even within the same tendon[2]. Kastelic et al.[65] measured the diameter of fascicles of 80-320 µm in mature rat tail tendon. Similar data were reported by Niven et al.[66] who observed collagen fascicles of 150- 300 µm in diameter. However, the number and diameter of collagen fibers vary considerably from tendon to tendon, ranging from 5 to 30 µm in the rat tail tendon[67] and being higher than 300 µm in human tendons[7].

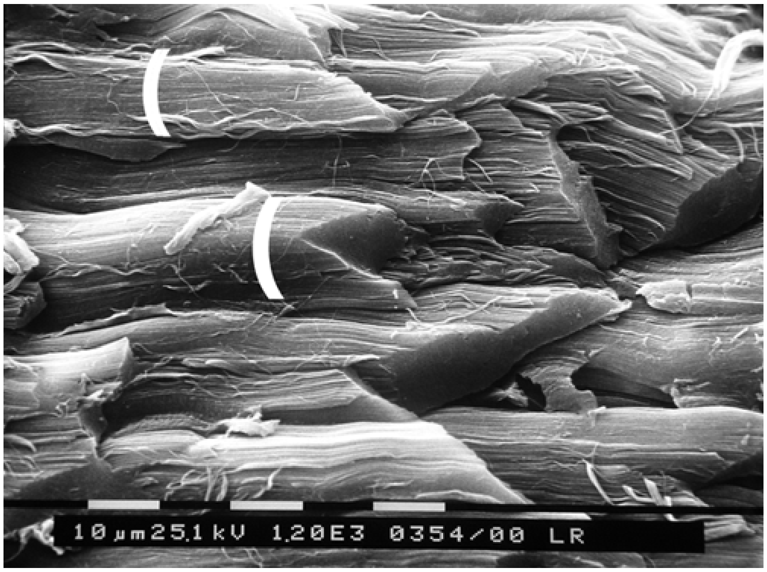

**FIGURE 7**. SEM image of rat Achilles tendon subfascicles (parentheses).
Bar = 10 µm

Tendon size also seems related to exercise, but the matter remains controversial. Although some authors[68,69,70,71] reported conflicting results in response to long-term training in tendons of different animals, Rosager et al.[72] showed that the cross sectional area of Achilles tendon in human runners seems to increase with long-term mechanical loading.

Many studies have investigated collagen alterations to tendons in old age, but the heterogeneity of different methods and experiments makes this topic unclear[73]. However, an *in vivo* study[74] reported that older tendons are 22% thicker than younger ones.

Some authors[75,76] showed that inside each fiber bundle, collagen fibrils are arranged in parallel fiber bundles running longitudinally but also transversally and horizontally, with longitudinal fibrils also crossing each other, forming spirals and plaits. Both the fascicles and tertiary tendon bundles frequently show a spiral arrangement along the course of the tendon[1]. Some studies favour the idea that collagen fibers are helically orientated in flexor bovine tendon and rat tail tendon[77]. In general there is a strong correlation between collagen orientation and functional requirements: tendon fiber direction is always indicative of the prevalent tensile stress[14].

## COLLAGEN FIBRILS

Tendon is composed of collagen fiber bundles each of them resulting from the aggregation of single collagen fibrils, namely tens of millions of collagen fibrils, each hundreds of microns long[16]. Collagen fibrils with a distinctive 67 nm axial periodicity are millimetres in length and range in diameter from a few nanometres to ~500 nm (depending on the tissue and stage of development)[9] (Fig. 8). Collagen fibrils are well stabilized by covalent intramolecular cross-links binding the collagen molecules to one another[6,78,79]. Both skeletal muscle stiffness and tendon cross-link concentration increase with aging, but endurance exercises counteract the increases[74,80]. Collagen fibril formation is a self-assembly process but is also sensitive to cell-mediated regulation[57]. In fact, fibril assembly seems to begin in the secretory pathway and at the plasma membrane of fibroblasts[9].

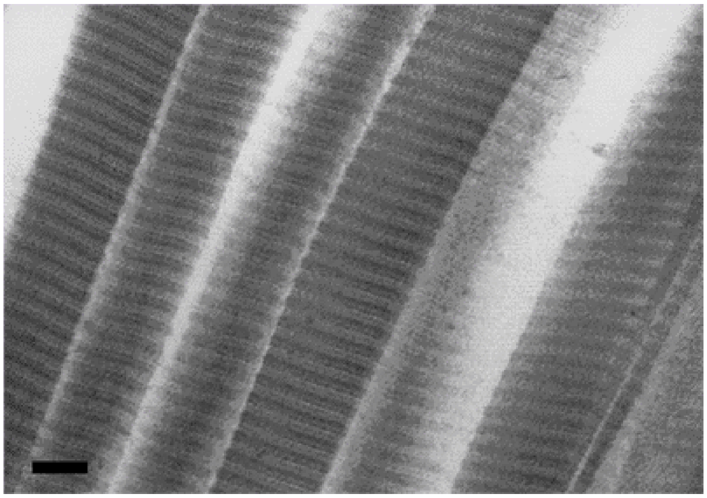

**FIGURE 8.** Electron micrograph of longitudinally sectioned flexor digitorum tendon of rat. Collagen fibers result from the aggregation of parallel straight collagen fibrils with a D-period and and a bimodal-plurimodal diameter. Inside each collagen fibril the almost straight arrangement of microfibrils is detectable.
Bar = 0,3 µm

Some authors observed that each single collagen fibril shows an inner structure or a subfibrillar arrangement of filamentous structures called microfibrils[81]. Collagen microfibrils range in size from 4 to 20 nm or more and show a straight or helical arrangement inside each fibril, depending on collagen fibril function[10,11,12,13,14,82]. In contrast with these considerations other studies failed to identify these microfibrils and supported the well-accepted fibril model of a quasi-crystalline structure[9,83,84].

The assembly of collagen fibrils in tendon is hierarchical and involves the formation of fairly short collagen early fibrils that are fusion precursors of the very long fibrils in mature tendons. In general, a subdivision of a single element into multiple, parallel threads of same cross-sectional area increases the safety factor and flexibility[14].

Collagen has an excellent elastic resilience. Some authors[85,86,87] suggested that the mechanical properties of tendons are related to the fibril diameter distribution and also that each collagen fibril, small and large, is likely to fulfil a defined functional role in tendon[14]. Smaller diameter fibrils are thought to be elastic and resistant to creep because of their larger total surface area that confers a higher interfibrillar interaction, while larger diameter fibrils are stronger because of an increased density of intramolecular cross-links. Fibril diameter increases during development[87,88] and decreases with aging [89] and disuse[90]. However the fibril diameter does not change along the tendon[91]. Inside a collagen fiber the collagen fibril diameter ranges from 20 nm to 150 nm[91,92] and even from 20 nm to 280 nm[59]. In human Achilles tendon the fibrils measure between 30 nm and 130 nm in diameter, while in the flexors and extensors of the fingers and toes they are 20-60 nm. The collagen fibrils run inside a collagen fiber prevalently parallel to each other in large straight bundles, but individual fibrils or bunches of them form spiral-plaits[76]. In tendons collagen fibrils show a bimodal or plurimodal diameter distribution including: 1) small diameter fibrils that ensure a better interfibrillar binding and creep resistance and 2) large collagen fibrils which are resistant to tensile strength[14] (Fig. 3). Considering the biomechanical properties of tendons, Silver et al.[93,94] reported that the ultimate tensile strength and the elastic modulus are more dependent on fibrils length than diameter. Ottani et al.[14] defined collagen fibrils in tendons as T-type collagen form, forming large, heterogeneous fibrils, parallel tightly packed and subjected to a tensile force along their axis. By contrast, collagen fibrils in sheaths surrounding tendons are considered C-type collagen form, consisting of small, homogeneous-unimodal fibrils, helically arranged and resisting multidirectional stresses[13,14]. In T-type large collagen fibrils the D-period is about 67 nm and they are built of collagen molecules lying at a slight angle ($< 5°$), while in small C-type collagen fibrils the D-period is 64 nm and the molecular pattern appears to be helical at a constant angle of 17°[95]. These aspects can explain why the fibrillar crimps we recently described[96,97] are detectable only in the large T-type collagen fibrils and not in C-type collagen (Fig. 4). Supporting this hypothesis, modeling studies suggest that fibrils diameters are inversely proportional to collagen molecular flexibility[98], so that the type III collagen molecule is more flexible than the type I collagen molecule[99].

## TRANSMISSION AND ABSORPTION OF FORCES IN TENDON

Collagen fibers act to transmit forces, dissipate energy and prevent mechanical failure in connective tissue[50]. *In vivo* studies in humans demonstrated that both static stretching of tendon and repeated muscle contractions resulted in a decrease of stiffness and an increase in the elongation of the tendon[100,101,102,103]. The mechanism underlying tendon elongation remains unclear. Some authors suggested that loading deformation of collagen fibers involves molecular stretching and slippage, followed by fibrillar slippage and ultimately defibrillation[50]. In particular, up to a macroscopic deformation of about 2% molecular stretching predominates[104] while beyond 2% D-period increase is the result of molecular slippage[104,105].

Stretching has been suggested to induce increased fibrillar orientation and packing density, leading to strong interactions within or between collagen fibrils[50,77,106]. Decorin, a small interstitial dermatan sulphate PG, is associated with the surface of fibrillar type I collagen and connects adjacent collagen fibrils in tendon. Decorin limits collagen fibril diameters by inhibiting the lateral fusion of fibrils[42,107] and inhibits the mineralization of fibrillar collagen matrices[42,108]. Decorin-type I collagen interactions appear to be stable in the absence of tensile stretching and improve the load to failure of collagen fibers.

*In vitro* studies showed that in the presence of tensile loads many of the collagen-decorin interactions are broken[50]. The authors suggested that decorin may be involved in promoting either molecular or collagen fibrillar slippage and improving lateral interactions during tensile deformations. Tendon viscoelastic properties are affected by decorin content but not by collagen alterations[109]. Cribb and Scott [43] suggested that forces are transmitted through the extracellular matrix of tendons via PG linkages between collagen fibrils proposing a sliding PG-filament model[110]. In particular the PG bridges were postulated to be orthogonally arranged between collagen fibrils in tendon, and may play a primary role in transmitting and resisting tensile stresses in tendons, contributing to the strength of the tissue. Supporting these data, Screen et al.[3,111] suggested that fiber sliding could provide the major mechanism enabling tendon fascicle extension within the rat tail tendon. Vogel[48] suggested that protective changes occurring in the structure of the tendon at the location of bending involve synthesis and accumulation of the large PG aggrecan. This accumulation protects tendon by providing compressive stiffness and allowing collagen fascicles to slide against one another. Moderate exercise for several weeks leads not only to a further increase in total proteoglycan content but also to qualitative changes in PGs make up [46].

On the contrary, Provenzano et al.[17] supported the idea that forces within tendon are directly transferred through collagen fibrils and not through an interfibrillar coupling such as the PG bridge. These authors observed that the collagen fibril ends, clearly visible in fetal tissues[16], were no longer present in either mature tendons and ligaments, collagen fibrils being either indeterminate in length or functionally continuous[9,86,112].

Another mechanism involved in transferring or absorbing forces in tendon during locomotion was supposed to be the collagen-crimping pattern. When observed at polarized light microscopy collagen fiber bundles show periodic light and dark bands which indicate a collagen wavyform arrangement or crimps, as described by many authors[29,30,66,76,113-121] (Fig. 9). The collagen fibers seem to have a planar zig-zag crimped configuration in rat tail tendon[65,66,116].

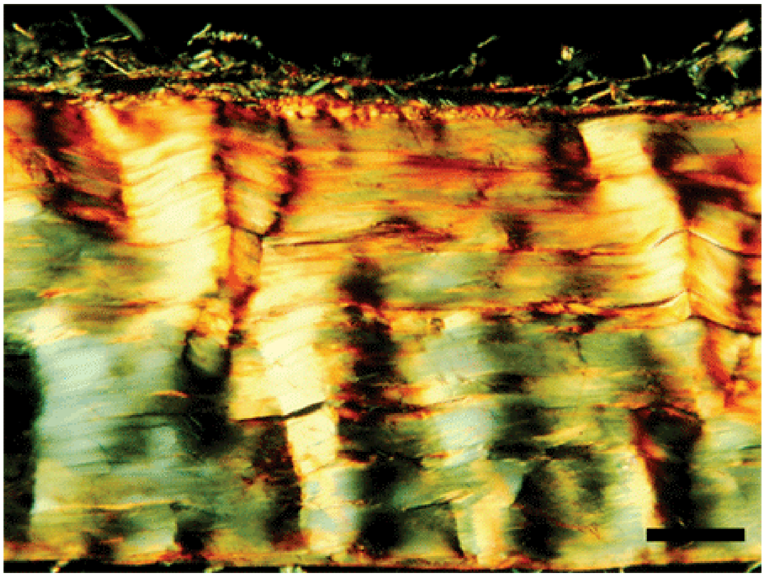

**FIGURE 9.** Polarized light micrograph of longitudinally sectioned rat Achilles tendon. Collagen fiber bundles show a crimping pattern characterized by alternating periodic dark and light bands. These structures correspond to the tendon crimps.
Bar = 100 µm

The crimp angle was investigated, varying between 0° and 60° [2,29,30] and decreasing slightly with age[115]. Crimp length was also measured, increasing until the animal reaches maturity[115]. Planar crimps were described into collagen fibrils perhaps by the contraction of cells at the ends of the fibrils or by shear stresses introduced by tendon cells between layers of collagen fibrils in developing tendons[8]. *In vitro* biomechanical studies demonstrated that tendon crimps disappear when tendons are slightly stretched[2,7,26,113,114,120]. When the tensile force is removed tendon again shows its normal crimping[113,114]. If the tendon is stretched up to 4% of its elongation the crimping pattern will not reappear[113,114], but tendon stretching was reported not to exceed 4% of its elongation during physiological joint movement[2]. The decrease of the number or flattening of crimps during tendon stretching seems to be related to straightening of the collagen fibers during loading[86,115,120]. In stretched tendons crimps disappear individually rather than simultaneously, from the ends toward the center of the collagen fascicles[120]. Some authors suggested that the initial nonlinear toe region of the stress-strain curve of tendon could be related to the straightening of tendon crimps with the disappearance of the light and dark bands[115,122]. Screen et al.[3] demonstrated that local strains within the collagen fascicle are smaller than the external applied strains, never exceeding 1.2% of axial strain, even at 8% gross applied strain. Tendon crimp has mainly been investigated from a histological point of view. Gathercole and Keller[117] studied collagen crimps in tendon by transmission electron microscopy without explaining their ultrastructure of crimps. Our recent study by atomic force microscopy, transmission and scanning electron microscopy showed that in relaxed rat Achilles tendon of rat the collagen fibrils suddenly changing direction within each tendon crimp show knots[96]. The fibrils appear partially squeezed in the knots, bent on the same plane, or twisted and bent (Fig. 10). Moreover some of them lose their D-period showing their microfibrillar component (Fig. 11). These particular aspects of collagen fibrils inside each tendon crimp have been termed “fibrillar crimps” and may fulfil the same functional role[97]. We demonstrated that when tendon is physiologically stretched *in vivo* the tendon crimps decrease in number (46.7%) ($p<0.01$) and appear more flattened with an increase in the crimp top angle (165° in stretched tendons vs. 148° in relaxed tendons) ($p<0.005$). At SEM and TEM, the “fibrillar crimps” are still present never losing their structural identity in straightened collagen fibril bundles of stretched tendons, even in areas where tendon crimps are not histologically detectable. It has been reported that type I collagen molecule largely abundant in tendons is rod-like with little flexibility and high mechanical strength[123], even if Silver and Birk[124] reported that the type I collagen has many bends and is not so rigid. These data supported the hypothesis that the fibrillar crimp in tendons

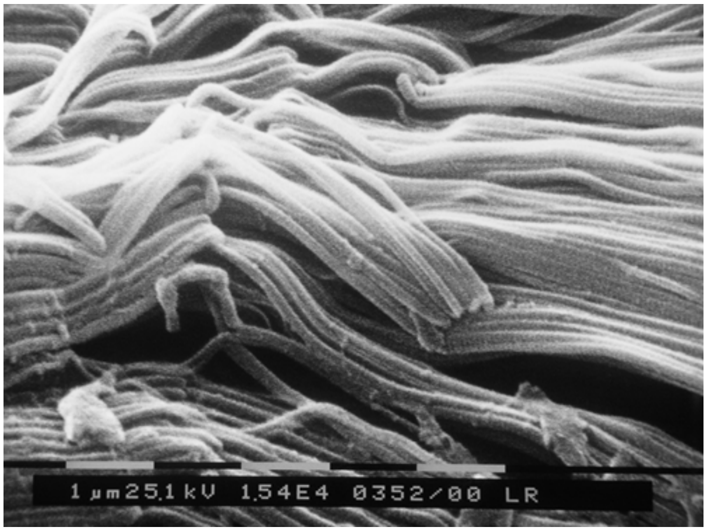

**FIGURE 10.** SEM image of longitudinally sectioned rat patellar tendon. Single collagen fibrils run parallel showing a D-period banding and have a bimodal-plurimodal diameter. Note fibrils suddenly changing direction, bending and appearing partially squeezed in knots.
Bar = 1 µm represents an ultrastructural molecular arrangement acting as a shock absorber during early tendon stretching[3,97,111,115,120]. Previous studies have suggested that mechanical energy translates into a molecular and fibrillar deformation increasing the collagen D-period[104,125,126]. However the elastic response seems to be mainly related to the end-to-end crosslinks between collagen molecules within the fibrils[93,94]. Other extracellular molecules seem to be related to the mechanical stretching of

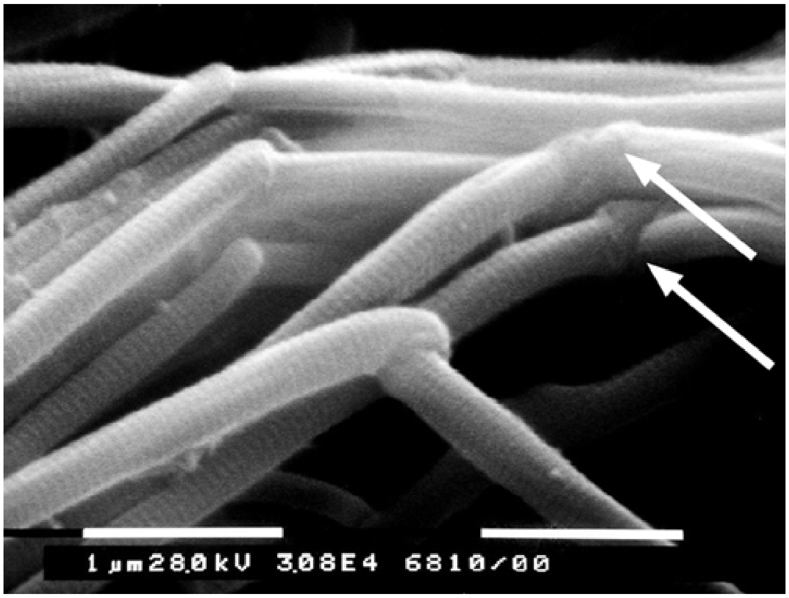

**FIGURE 11.** Electron micrograph of longitudinally sectioned rat tail tendon. Where fibrils change their direction, they loose their D-period (arrows). These knots have been termed "fibrillar crimps".
Bar = 1μm tendons: it was reported that the production of tenascin-C and collagen XII, closely associated to type I collagen fibrils[127], is high in fibroblasts attached to a stretched collagen matrix, but suppressed in cells on a relaxed matrix[128]. The response to a change in stretch is rapid and reversible and is reflected at the mRNA level. Type XII collagen acts to decrease the interactions between collagen fibrils then allowing them to slide past each other when an external force is applied[16,129].

To better understand the mechanisms and tendon components involved in stretching and transmission of tensile forces within tendon, it is important to remember that tendon function is related to aging. Younger tendons break at lower stresses than mature ones[43]. In fact older tendons are less capable than younger tendons of transmitting fast forces from muscles to bones, and they are subjected to smaller tensile stresses than younger ones[73,74]. Aging is associated with: 1) an increase in non-reducible collagen cross-linking, 2) a flattening of collagen crimps, 3) an increase in elastin, 4) a reduction in extracellular water and PGs, 5) an increase in type V collagen[73,130,131,132]. It is evident that changes in collagen cross-links, crimp morphology and PGs content in tendon are related to mechanical properties of tendon in transmitting tensional forces.

There is still no complete understanding of the mechanisms in transmitting tensional forces within the tendon *in vivo*. However it seems probable that at a first stage of tendon stretching, with very slow forces, a flattening of tendon crimps may occur. Increasing the stretching, but still at small strains, a straightening of molecular kinks of collagen fibrils may happen. Finally, higher strains lead to molecular gliding within the fibril structure. However, as the strain within collagen fibrils is only 40% of the strain applied on the rat tail tendon, it is reasonable that an additional gliding process occurs at the interfibrillar level[86].

In conclusion it is probable that tendon stretching and transferring forces within tendon occur a) by flattening of tendon and fibrillar crimps, b) through an interfibrillar coupling via PG linkages, and c) directly through collagen fibrils.

## ACKNOWLEDGMENTS

We are indebted to Gianfranco Filippini, D.I.S.T.A., University of Bologna, for his technical assistance with SEM. This work was supported by MIUR Grant (prot. # 2004055533).
